# The Ward Laboratory.—II

**Published:** 1912-05-18

**Authors:** 


### THE MAKING OF A MODERN HOSPITAL.*

### The Ward Laboratory.—II.

The ward laboratory is usually placed in one of the annexes to the ward itself. Whether this is in the centre or at the end of the pavilion does not very much matter; the essential points are that the laboratory must be conveniently situated, not too close nor too far removed from the ward, that it must be well ventilated and lighted, and that it must be private, so that, if necessary, it can be shut off from the ward itself and from the other annexes. It is essential that it should be close to the ward itself, for, as already explained, the investigations which are to be carried on in it are bedside examinations; they must be made on the spot with as little delay as possible. At the same time the patients in the ward must not be inconvenienced by the work that goes on in the ward.

### An Important Point.

This is a point that needs some consideration. The simple process of boiling a test-tube of urine may, if the laboratory is actually part of the ward, become an unpleasant and unwholesome process so far as the patients and workers in the ward are concerned. Even more necessary it is to ensure quiet and calm in the laboratory. For that reason it is desirable that the laboratory should be placed in a separate room, and it is convenient, in pavilions where provision is made for a physician's or surgeon's room, to utilise this apartment for the laboratory. In all institutions the resident staff have adequate accommodation centrally; if a sitting-room is provided for the house physician or surgeon in each pavilion or ward unit, it may justifiably be termed the work-room and used as a laboratory. In the general plan of the ward already given in a former article (The Hospital December 9, 1911, p. 257) this alternative has been kept in view, and the special apartment designed for the use of the staff has been marked "Ward Laboratory."

It is desirable also that the laboratory room should be well isolated from the ward kitchen, the day-room (where the unit contains one) and the nurses' or sisters' room. It is also preferable that it should have no direct entrance to the ward itself, but be entered by a lobby or corridor door; this is to ensure privacy and guard against the entrance of patients—a point of considerable importance, for not only is delicate and easily disarranged apparatus kept in the laboratory, but among the reagents used are certain poisons and dangerous solutions which should not be within reach of patients. It is not always possible to keep these reagents apart in a special poison locker or cupboard, but care should be taken that the ward laboratory, when it is not occupied by some responsible person, is kept locked.

Adequate lighting and ventilation must be provided for, and the laboratory must be efficiently heated. Lighting is best arranged for by giving at least one direct window light, although, where possible, two or even three sides of the room should be provided with windows; this ideal arrangement can, of course, only be secured by having the rooms allotted to the laboratory built out from the ward or annexe—an arrangement which for various reasons is unlikely to be popular. In the circumstances a single or double window light must suffice, but ample light may be gained by making the windows very long so that they reach almost from floor to ceiling; here, as elsewhere in the unit, double windows are desirable. As a great deal of the laboratory work will be carried out by artificial light, care must be taken that the burners or lights are so arranged that they give the fullest brilliancy without undue glare.

Most modern ward laboratories are lit by electric light, metal filament lamps being employed; those pendent from the ceiling should be in plain-glass globes, and those used for microscopic work in opaque, or partly opaque, globes. It is as well, however, to have an incandescent gas-burner in case of emergencies, since gas must also be used for the Bunsen burners; it is true some very modern ward laboratories rely entirely on electricity for lighting, heating, and for the working of the apparatus, but the simple, old-fashioned Bunsen burner is, after all, to be preferred to the elaborate electric heaters, so far as boiling of fluids in test-tubes or drying of films is concerned. Ventilation must be secured by natural means. For heating an open coal fire is the best; where that cannot be secured a gas fire serves admirably. If the unit is centrally heated, radiators will of course be provided; but even in that case the provision of an open fire adds greatly to the worker's comfort. Special provision must be made to ensure the adequate exit of any combustion or evaporation vapours; an evaporating chamber of the type used in a chemical laboratory is hardly ever necessary, but a small evaporating chimney, communicating with the chimney of the fireplace, is a great convenience. This is cheaply constructed of block tin and arranged over the working bench; it can be painted or enamelled to harmonise with the walls.

### Floor, Ceiling, and Walls.

The ceiling, flooring, and walls of the laboratory need no special attention; the keynote here, as elsewhere in the unit, should be simplicity. A tiled floor, with the tiles reaching halfway up the wall, the corners rounded and all joints made flush, is generally satisfactory; a good wood floor, or a linoleum on cement flooring does just as well;

* Previous articles appeared on Oct. 7, 14, 21, 28, Nov. 4, 18, Dec. 9, 30, Feb. 10, March 16, April 6, 20, & May 4.

composition and rubber floorings should be avoided, as they are specially apt to be stained by the dropping of chemicals and aniline dyes.

The furnishing of the ward laboratory is a matter of considerable importance. It is always desirable to get the very best material in the way of apparatus and fittings; this ensures good results and is in the end the most economical. First, and perhaps most important, is the water-supply. A wash-basin, with hot and cold water, for the use of the laboratory workers, a large sink also provided with hot and cold water taps, and a smaller one, with a large trap for washing infected tubes, are necessary. Both sinks should have wire guards over the exits so as to prevent blockage by glass, fragments of tissue or *débris*; the sinks must be arranged at a convenient distance from the wash-basin. Smaller sinks must be provided for the tables or benches. Of these at least two must be provided: a table or bench for bacteriological and microscopic work, and one for chemical tests and investigations. These benches or tables should be of the usual laboratory pattern; for the bacteriological bench the black-glass surface, as employed at the Charing Cross Hospital laboratory, is very suitable. One or two "places" are in general all that are required at each bench. The reagents and apparatus which may be required should be stored in locked, glass-fronted cupboards or shelves, immediately above the tables, while ample locker accommodation must be provided below the benches. The arrangement of places which has been adopted at the post-graduate laboratory in the Luisenplatz at Berlin is perhaps the most convenient; it ensures facility of movement on the part of the worker with economy of space; the arrangement of the lamps is particularly excellent. A large cupboard must be provided for overalls and towels; this is conveniently placed near the door and some distance away from the sinks and benches.

### Bench Equipment.

The equipment of the benches will vary according to the nature of the work that is likely to be done in the laboratory. In the ward laboratory what is to be done is the investigation of body fluids, excreta, and possibly tissues, in case a quick decision is desired as to the character of a portion of tissue excised in the course of an operation. The bacteriological bench will, therefore, hold the usual arrangements for staining, fixing, and examining slides; an incubator, for the use of quick cultures, as for the investigation of suspected cases of diphtheria, is hardly necessary or advisable. A centrifuge must be provided on this as on the chemical table; a small hand-power machine is usually sufficient, but some recent models, especially made for the ward laboratory, are driven by electricity, and are very compact, easily handled, noiseless, and reliable; the only objection to their use is that they are somewhat expensive.

A good microscope must be provided. This is preferably kept under a bell jar, with a triple nose piece holding the usual three powers—$\frac{1}{4}$, $\frac{1}{6}$, and $\frac{1}{12}$; the instrument is always ready for use. Here, again, a good type of instrument is preferable to a cheap one; the expensive Leitz and Zeiss microscopes are admirable, but for ward work an instrument by a first-class English firm, such as Watson or Ross, will be found quite suitable. Apparatus for blood examinations, including a good type of hæmoglobinometer and hæmocytometer, must be provided. Nowadays, where a quick serum diagnosis is often of the greatest service, additional apparatus will be required for conducting the usual serum tests.

### Chemical and Microscopic Apparatus.

The chemical side of the laboratory will be provided with the usual assortment of apparatus for carrying out the ordinary chemical tests demanded in ward work. Burettes and estimating flasks must be conveniently arranged, and care should be taken that they are kept in good order and that the reagents are fresh. More elaborate apparatus, such for instance as are used for cryoscopy, are usually relegated to the larger laboratories; if they are included in the ward laboratory's outfit they must have a special table or bench. Similarly if tissue examinations are to be made, a stand for a microtome and for the preparation of the slides, for embedding and cutting the sections, must be provided. The microscope on the bacteriological table will serve the three departments equally. There is no reason to duplicate apparatus for it is hardly likely that two examinations requiring the use of the same instrument at the same time will be carried on simultaneously. Simplicity in the arrangements is of the greatest importance, and everything should be done to keep the ward laboratory as simple as is consistent with efficiency.

It need scarcely be remarked that such a well equipped, well arranged modern ward laboratory needs proper attention and care. It should be the duty of the house surgeon or physician, or whichever member of the staff is in charge of the ward unit to satisfy himself everyday that the room is kept in proper order, clean and orderly and that everything is put back in its place after it has been used.

If possible no experiments or investigations should be prolonged over night; if an examination demands so much time it should be relegated to the institutional laboratory and not carried out in the ward laboratory. When an investigation necessitating the employment of infected material has been made, care should be taken that the contents of test tubes, the slides, etc., are properly sterilised and disinfected before they are thrown into the sink or into the refuse box; no infected material should be allowed to enter the drains. The cleaning of the laboratory is generally entrusted to the wardmaid under the superintendence of the ward sister or head nurse. In cases where there is a central laboratory, it is a good plan to get the laboratory boy or attendant to visit the ward laboratories once or twice daily and see that everything is in its proper place.

